# Teaching communication skills to OTL-HNS residents: multisource feedback and simulated scenarios

**DOI:** 10.1186/s40463-019-0329-8

**Published:** 2019-01-28

**Authors:** Pier-Luc Beaudoin, Mathilde Labbé, Amanda Fanous, Meredith Young, Jamie Rappaport, Yoon Soo Park, John Manoukian, Lily H. P. Nguyen

**Affiliations:** 1 Faculty of Medicine, McGill University, Montreal, Quebec Canada; 2 Department of Otolaryngology-Head and Neck Surgery, Montreal Children’s Hospital, McGill University, 1001 boul. Decarie, A02.3015, Montreal, Quebec H4A 3J1 Canada; 3 Center for Medical Education, McGill University, Montreal, Quebec Canada; 4 Department of Medicine, McGill University, Montreal, Quebec Canada; 5 Department of Medical Education, University of Illinois, Chicago, Illinois USA

**Keywords:** Communication, Communication skills, Simulation-based teaching, Multisource assessment, Medical education

## Abstract

**Background:**

Effective communication has been linked to a reduction in adverse events and improved patient compliance. Currently in Otolaryngology – Head and Neck Surgery (OTL-HNS) residency programs, there is limited explicit teaching of communication skills. Our objective was to implement an educational program on communication skills for residents using multisource assessment in several simulation-based contexts throughout residency.

**Methods:**

For three consecutive years, OTL-HNS residents were recruited to participate in a total of nine simulation-based clinical scenarios in which communication skills could be honed. This educational program was designed to provide instruction and practice of challenging ethics scenarios, with communication efficacy as a secondary goal. To facilitate this goal, a multisource assessment was paired with a debriefing process that involved attending staff, observing and participating residents, standardized patients, and invited content experts.

**Results:**

Seventeen residents completed the curriculum for at least two consecutive years from 2009 to 2011. The internal-consistency reliability of the scenarios ranged from 0.88 to 0.96. The intraclass correlation was 0.19, as expected in this context. There was no statistical difference in the mean ratings of performance across post-graduate year (PGY) level (*p* = 0.201). Results from the random-intercept regression indicated that, on average, a learner’s mean rating at baseline was 3.6/5 and increased significantly by 0.25 points per year (*p* < 0.05) as assessed by OTL-HNS staff members and peers. No significant improvement across time was found for ratings by non-medical assessors.

**Conclusion:**

Implementing an educational program focused on communication skills using a multisource assessment in various contexts has shown to be potentially effective at our institution, and resulted for yearly improvement and consolidation of performance of OTL-HNS residents as judged by faculty and residents. The inclusion of a multisource assessment in the simulation curriculum is key to allow for the representation of different perspectives on communication skills, for both the assessment and the debriefing process. Future studies are needed to explore the possibility of fully integrating this educational program into residence training in order to support deliberate communication skills teaching.

## Introduction

Communication is a key element of any physicians’ practice – necessary for both patients and colleagues. The ability to share information effectively has been linked to improved patient adherence [1] and failure to do so has been shown to be one of the leading causes of adverse events [2, 3]. There is also increasing evidence of the positive effects of communication skills training on both physicians’ and patients’ overall well-being [4–6].

Within the Otolaryngology-Head and Neck Surgery (OTL-HNS) residency training program, there is often an assumption that residents develop communication skills implicitly as they progress through their training, and there are few formal teaching opportunities centered on effective communication skills in most residency programs [7, 8]. There is, however, growing evidence that some communication skills are not spontaneously acquired through clinical experience alone [9, 10]. There is also growing consensus that communication is a skill that can be taught [5] and recent literature has documented the benefits of communication skills teaching as part of residency training in other fields of medicine [10–12]. Effective communication is challenging and performance varies greatly depending on context; a phenomenon often referred to as context, or content, specificity [13]. For instance, an individual that is skilled at delivering bad news to a patient’s family may not necessarily be as skilled at clearly describing the risks and benefits of a surgical procedure.

In order to facilitate the development of the skills necessary to communicate effectively, and to permit opportunities to practice these skills, we designed an educational program consisting of a longitudinal simulation-based ethical-legal curriculum designed specifically for OTL-HNS residents. Within this educational program, we emphasized the formative assessment of communication skills across a variety of contexts relying on multisource assessment from various evaluators (non-medical observers, peers, faculty, standardized patients and self). The design, evaluation, and outcomes of the ethics component of the educational program have been previously published, and are available elsewhere [14]. The aim of the work reported here is to explore the evolution of residents’ communication skills across the longitudinal training, through the lens of multisource assessment and feedback.

## Methods

Approval from the institutional ethics review board at McGill University (Montreal, Canada) was obtained prior to the start of this prospective study.

### Simulation setting

**Fig. 1:**
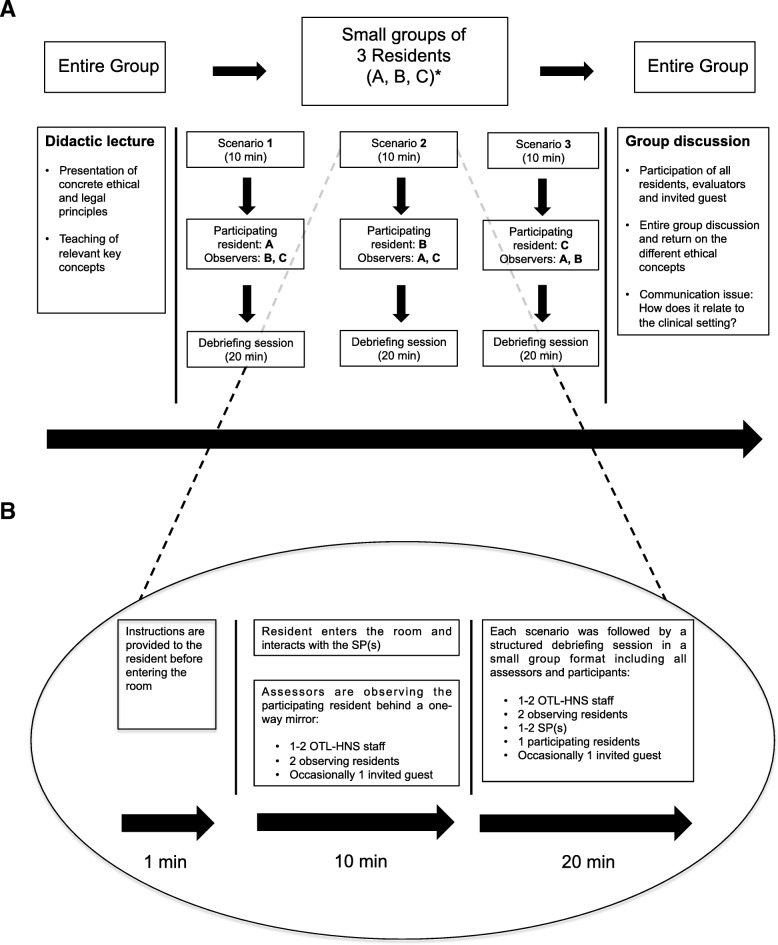
**a** Program of the yearly half-day course covering three ethical-legal scenarios; **b** Organization of the ethical-legal simulated scenarios

The evaluation of communication skills was a secondary objective of a longitudinal simulation-based ethical-legal curriculum that was developed for OTL-HNS residents at our institution. The design, content, and evaluation of the ethics component of this educational program has been reported elsewhere [14]. To briefly summarize, the curriculum consisted in yearly half-day courses spanning three consecutive years of residency. Each half-day course was comprised of three 10-min simulated scenarios using standardized patients (SP) during which residents were challenged with a difficult ethics scenario before joining a 20-min debriefing session in small group format. (Fig. 1a) Each resident participated in one scenario, and observed their peers in two others. The team of evaluators had access to examining rooms with one-sided mirrors for the simulated scenarios.

During these scenarios, the primary objective was the application of stage-appropriate ethical-legal knowledge, and the secondary objective was the demonstration of communication skills. Each simulated scenario was constructed to recreate clinically relevant situations necessitating both application of ethics or legal principles, and demonstration of communication skills. Residents rotated through three 10-min scenarios that were each immediately followed by a small group debriefing session. (Fig. 1b) In the debriefing, the instructor or faculty member would provide feedback on how to improve communication skills.

For three consecutive years, all Otolaryngology residents (PGYs 1–5) from our institution were required to attend the yearly half-day course and were subsequently invited to participate in the study. Participation in the study included consent to release assessment data for the purposes of analysis. All residents were welcome to participate in the educational program, regardless of participation in the research study. Data presented here reflect those residents who provided consent for data analysis for at least two consecutive years. In this context, data recorded for PGY-5 s from the first year of the curriculum and PGY-1 s of the third year of the curriculum could not be included in the study.

### Multisource assessment and *Debriefing Methods*

An institution-specific tool was developed based on the Accreditation Council for Graduate Medical Education (ACGME) Educational Resource to improve communication skills [15]. Task-specific items were included depending on the specific content of the simulated scenario. The assessment tool included seventeen items relevant to communication skills, each rated on a five-point Likert scale (anchored from Poor to Excellent), and eight to thirteen items were completed for each scenario depending on the applicability of individual items within the scenario [16]. Rater-training was provided to all assessors.

For each scenario, the same communication skills assessment tool was completed by medical evaluators which included: the participating resident (self-assessment), two observing residents (peer-assessment) and the OTL-HNS staff(s) (faculty assessment). Additionally, the assessment tool was also completed by non-medical evaluators that included: standardized patients (patient assessment) and occasionally a rotating invited lawyer/ethicist (content expert assessment). The language used in the assessment tool consisted of non-medical terms, in order to engage all assessors, including those with non-medical backgrounds as previously stated.

There were two different opportunities for debriefing during the half-day course. At the end of each scenario, all of the evaluators attended a debriefing session and were encouraged to provide specific feedback to the participant. Furthermore, a second debriefing session was organized at the end of the half-day course to allow participants to discuss specific points that they thought was pertinent for the entire group.

The same six OTL-HNS staff consistently served as assessors for all years of the curriculum. They were chosen based on their ability to give appropriate and targeted feedback to residents. Consistency of assessors’ debriefing methods was accomplished by pre-assigned readings on debriefing points and yearly faculty debriefing sessions held prior to the courses. All assessors used the Pearls Healthcare Debriefing Tool [17].

### Data analysis

Data were collected over three consecutive years (academic year of 2009–2010 to 2011–2012) and analyzed for this study. Descriptive statistics were performed on participant characteristics. Cronbach’s alpha was calculated to evaluate internal-consistency reliability. The Intraclass Correlation (ICC*)* was used to calculate the inter-Rater Reliability. Independent t-tests for mean differences in assessment ratings from all assessors across reported genders were used. Analysis of variance (ANOVA) was used to compare mean ratings from all assessors across PGY levels. Random-intercept regression was used to examine whether learner performance improved over time (2009 to 2011). Ratings from medical (faculty and peer) and non-medical (standardized patient and content expert) assessors were also analysed separately to determine if there was a difference in performance rating over time between the two groups. Finally, the mean score of all participants for each item of each station was analyzed to identify strengths, weaknesses, and areas of variable performance for the study cohort. We defined items as *poorly performed* if the mean score was at least one standard deviation below the mean score of all items for each station. We defined items as *variable* if the standard deviation (SD) of all scores for that item were more than one SD above of the mean SD of all items for each station. All data were presented on an average out of a score of 5 and a perfect score was considered to be 5/5.

## Results

A total of 28 residents participated to the curriculum from 2009 to 2011. Out of those 28, 17 residents participated in this study for at least two consecutive years, with each separate evaluation included in this study. There were no residents that refused participation in the study, therefore eliminating a potential selection bias. As seen in Table 1, the number of items completed for each station varied from eight to 13. The inter-item correlation of each of the nine scenarios ranged between 0.42 and 0.76, and the results of the internal-consistency reliability (Cronbach’s alpha) ranged between 0.88 and 0.96. The ICC was 0.19 (95% CI = 0.06, 0.49) thus showing a low consistency of performance between scenarios.

**Table 1:** Reliability of the scenarios

Year	Scenario	Communication Scale		
		# of Items	Inter-Item Correlation	Reliability
1	1: flirtatious patient	10	.42	.88
	2: research ethics	10	.42	.88
	3: aggressive patient	8	.76	.96
2	4: disclosure	13	.45	.91
	5: resources management	13	.46	.92
	6: incompetent patient	13	.64	.96
3	7: euthanasia	12	.46	.91
	8: privacy/confidentiality	13	.48	.92
	9: impaired physician	11	.52	.93

There was no significant difference in mean communication score across genders (*p* = 0.119). The initial score of all participants on entering the curriculum, regardless of PGY level, was not statistically different (*p* = 0.201).

The random-intercept regression, which comes from the score provided by all of the evaluators including self, showed the learner’s overall mean communication score to be 3.60 at baseline with an increase of 0.25 points per year (95% CI: 0.10, 0.40) (*p* < 0.001). The results of the multisource assessment also showed that learners that had initial difficulty with communication skills (as indicated by their score on the first year of the module) improved enough to reach the average of their peers’ performance on the subsequent years.

Results showed variability in assessor ratings over time. Mean ratings from medical observers (OTL-HNS staff, observing residents) were 3.67 and increased by 0.31 points per year (*p* < 0.001). Non-medical observers (lawyer or ethicist and standardized patients) mean ratings were 3.60 with an increase of .06 points per year (*p* = .721).

The item specific analysis revealed items that were consistently poorly performed, or variably performed by residents (Table 2). Data are presented by scenario, suggesting the scenarios in which a given skill was either poorly performed, or had a variable performance.

**Table 2:** Scenarios where the communication skill was poorly performed and where the performance was variable amongst residents

Communication Skills	Poorly Performed	Variable Performance
1. Uses appropriate body language and maintains eye contact		
2. Effectively listens to concerns and perspective of patient	Scenari 3	
3. Allows patient the time to express his/her concerns		
4. Acknowledges, explores and understands concerns of the patient		
5. Picks upon clues, both verbal and non-verbal	Scenari 3, 5	Scenario 7
6. Pays attention to transitions	Scenari 5	Scenario 4
7. Uses open and closed questions effectively	Scenari 4, 7	
8. Directs the interview effectively		
9. Remains professional with the patient		
10. Summarizes, provides closure and an opportunity for questions	Scenari 1, 2, 6, 7, 8, 9	Scenario 2,3,8
11. Demonstrates empathy and support	Scenari 7, 8	Scenario 2
12. Demonstrates professional behavior and treats the patient with courtesy and respect		Scenario 5
13. Negotiates with the colleague and tries to come to mutual agreement	Scenari 6	Scenario 2, 6
14. Verifies understanding of situation by the colleague	Scenari 2, 5	Scenario 6, 8
15. Keeps voice at a conversational level		
16. Greets patient and asks what he wishes to discuss		
17. Shares information using language that patient can understand		Scenario 8

## Discussion

To our knowledge, this is the first report of and educational program designed to teach and assess communication skills relying on multisource assessment within simulation-based contexts for OTL-HNS residents. Residents were assessed formatively by peer residents, attending staff, standardized patients, content experts, and engaged in self-assessment using locally developed assessment tool. The assessment tool showed an internal-consistency reliability ranging from good to excellent with values between 0.88 and 0.96 thus providing evidence of reliability. A result of 0.19 for the ICC shows that the stations were highly variable in terms of content, as intended, but were able to differentiate across resident ability.

The initial score of all participants on entering the curriculum, regardless of PGY level, was not statistically different. This means that the most senior residents at the start of the curriculum were assessed to have similar communication skills as the most junior ones. From this curriculum, we raise the question as to whether the current model of residency training in OTL-HNS where communication skills are assumed to be indirectly acquired through clinical practice, and are not explicitly taught, is adequate. There is therefore evidence to support the integration of formal and explicit teaching of communication skills, including opportunities where OTL-HNS residents can practice their communication skills and receive feedback.

Scenarios included in our educational program were designed to assess different aspects of effective communication. The relatively low ICC, which is a measure of the consistency of performance between scenarios included in our educational program (0.19), was an expected result. This low ICC is similar to what has been previously shown in the literature around the Objective Structured Clinical Examination (OSCE) [18] and reinforces the notion that effective communication incorporates a broad range of skills and the demonstration of these skills can be context dependent [19]. Therefore, a resident that performed well in one scenario may not perform well in another. Context dependence of performance can also be seen for specific skills across scenarios.

Item-level analysis uncovered skills that were poorly performed, or highly variably performed – but these skills differed depending on the content of the scenario itself. Meaning that the items for which the residents were less successful overall and performed more variably, differed from one scenario to the other; demonstrating that there was variability of performance of the same communication skill across different contexts. Communication training within a residency program should therefore allow for exposure, practice, and feedback opportunities across many types of settings, highlighting the application of different communication skills in a variety of contexts.

Aggregating scores across all assessors, the mean score of residents’ performance increased steadily every year. This suggests that our simulation-based teaching is effective*,* regardless of PGY level. Residents in their first year of the educational program showed greater variability in performance, however we see convergence of performance across time. This suggests that all residents attained a target minimum acceptable performance similar to that of their peers by the end of the educational program, including residents who began with the poorest scores. A choice was made not to compare the curriculum to a control group, as we felt that all residents from our program should take advantage of such a curriculum. As such, one limitation of our study is the inability to control for improved communication skills due to extrinsic environmental factors as the residents progressed in their training.

The inclusion of multisource assessment in the simulation curriculum allowed for the representation of different perspectives on communication skills, both in the assessment and debriefing process. Including peer assessment allowed for non-participating residents to engage throughout the session and become familiar with the assessment tool (reinforcing the target communication skills), therefore contributing to the educational value of the program. In contrast with the clinical setting, where there is very little opportunity for patient feedback [20], the debriefing process including standardized patients allowed for a more complete formative learning experience.

In deconstructing the potential perspectives within our assessment data, we explored assessment patterns for medical, and non-medical assessors. We found that ratings generated by medical observers (OTL-HNS staff and observing residents) increased over time, whereas ratings from the non-medical observers (lawyers/ethicist and standardized patients) did not. The difference between medical and non-medical observers in ratings of communication skills across time should give us pause. One potential explanation of this discrepancy is the possibility that the characteristics of good communication may differ across these two groups of raters. This could imply that taking into account the perspective of the patient when teaching communication skills could enrichen the educational experience and provide different kinds of feedback to support improved performance [21]. An alternate explanation for this discrepancy of results is what is called the halo effect. In the halo effect, the assessor’s judgment of a candidate’s performance may be biased due to the assessor’s prior impression of the candidate. Since trainees are assessed throughout all aspects of residency by attending staff that know them, the above explanation could imply that evaluation of performance, or progress, may sometimes be overestimated. One could wonder if there is a need for more regular evaluations from blinded assessors during residency, however this remains an area for future research. The differences in change in scores across time by the two groups of raters may require a nuanced investigation regarding the objective efficacy of our educational program in improving residents’ interactions with patients. The distinctions between the assessment patterns for the medical and non-medical observers also raises questions regarding the ways in which assessment data should be consolidated when that assessment data is drawn from different sources - particularly when data support different conclusions regarding performance. Given the formative nature of the multisource assessment used here, no high-stakes decisions were made based on this data. However, further research is needed to better understand differences in assessment scores, and to explore how best to combine multisource assessment scores in order to best support resident learning.

The primary limitation of this study is that the assessment tool used to evaluate communication skills has not undergone a formal validation process. However, there is evidence presented in this study, including high internal-consistency and longitudinal growth over time by raters, which underscore the utility and reliability of the scores generated by the assessment tool. Efforts are underway to conduct a more comprehensive validity study that integrates the curriculum within the residency program. Furthermore, our participants were not formally compared to a traditional control group, as we believed that all residents from our institution would benefit from communication skills training, and progress across time would demonstrate the utility and benefit of the educational program. Finally, as the medical assessors (residents and attending staff) were all familiar with the participants throughout the years, there could have been an assessment bias, possibly contributing to the discrepancies seen in our results between medical and non-medical raters.

## Conclusion

Teaching communication skills across various contexts using multisource assessment could be effective in improving the performance of OTL-HNS residents. Future studies are needed to extend the validation process of the assessment tool and to explore the possibility of fully integrating this educational program into residence training in order to support deliberate communication skills teaching.

## Abbreviations

ACGME: Accreditation Council for Graduate Medical Education
ANOVA: Analysis of variance
ICC: Intraclass Correlation
OSCE: Objective Structured Clinical Examination
OTL-HNS: Otolaryngology-Head and Neck Surgery
PGY: Postgraduate year
SD: Standard deviation
SP: Standardized patient
